# Role of L-ascorbate in alleviating abiotic stresses in crop plants

**DOI:** 10.1186/1999-3110-55-38

**Published:** 2014-04-09

**Authors:** Jelli Venkatesh, Se Won Park

**Affiliations:** Department of Molecular Biotechnology, Konkuk University, 1, Hwayang-dong, Seoul, Gwangjin-gu South Korea

**Keywords:** Abiotic stress, Antioxidant, L-ascorbate, Reactive oxygen species, Transgenics

## Abstract

L-ascorbic acid (vitamin C) is a major antioxidant in plants and plays a significant role in mitigation of excessive cellular reactive oxygen species activities caused by number of abiotic stresses. Plant ascorbate levels change differentially in response to varying environmental stress conditions, depending on the degree of stress and species sensitivity. Successful modulation of ascorbate biosynthesis through genetic manipulation of genes involved in biosynthesis, catabolism and recycling of ascorbate has been achieved. Recently, role of ascorbate in alleviating number of abiotic stresses has been highlighted in crop plants. In this article, we discuss the current understanding of ascorbate biosynthesis and its antioxidant role in order to increase our comprehension of how ascorbate helps plants to counteract or cope with various abiotic stresses.

**Electronic supplementary material:**

The online version of this article (doi:10.1186/1999-3110-55-38) contains supplementary material, which is available to authorized users.

## Review

### Introduction

Adverse environmental factors such as excessive cold, heat, drought and salinity stresses result in a considerable yield loss of crop plants all over the world. These abiotic stresses elicit complex cellular responses in the plant system, resulting in the production of excessive reactive oxygen species (ROS) such as hydrogen peroxide (H_2_O_2_), hydroxyperoxyl (HO_2_**·**), superoxide (O_2_^-^) and singlet oxygen (^1^O_2_) radicals. Excessive ROS generated in plant cells tends to interact with different macromolecules resulting in oxidation of proteins, membrane lipids and nucleic acids and causes cellular damage, ultimately affecting the growth and productivity of plants (Wang et al. 2003). To protect themselves from adverse conditions, plants have evolved a number of cellular defense mechanisms including antioxidants such as ascorbate, glutathione and tocopherols as well as ROS-detoxifying enzymes such as superoxide dismutases, peroxidases and catalases (Inzé and Van Montagu 1995; Noctor and Foyer 1998).

Among the plant antioxidants, L-ascorbate is a major antioxidant playing a vital role in the mitigation of excessive ROS activity through enzymatic as well as non-enzymatic detoxification (Mittler 2002). It also acts as a cell signaling modulator in numerous cellular processes including cell division, cell expansion and cell wall growth (Liso et al. 1984; Conklin and Barth 2004; Wolucka et al. 2005; Zhang et al. 2007). It is a cofactor for the number of enzymes such as violaxanthin de-epoxidase (VDE, xanthophyll cycle), 1-aminocyclopropane-1-carboxylic acid (ACC) oxidase (ethylene biosynthesis) and 2-oxoacid-dependent dioxygenases (ABA and GA biosynthesis) (Eskling et al. 1997; Davey et al. 2000; Smirnoff 2000). Plants with low ascorbate biosynthesis are rather sensitive to various environmental stress conditions affecting their growth and development (Müller-Moulé et al. 2004; Huang et al. 2005; Alhagdow et al. 2007; Gao and Zhang 2008). Recently, it has been reported that ascorbate plays a crucial role in protection against various environmental stresses such as, drought (Hemavathi et al. 2011; Fotopoulos et al. 2008), salinity (Kwon et al. 2003; Huang et al. 2005; Wang et al. 2005; Sun et al. 2010a; Zhang et al. 2011; Venkatesh et al. 2012), ozone (Zheng et al. 2000; Sanmartin et al. 2003; Feng et al. 2010), low/high temperatures (Kwon et al. 2003; Larkindale et al. 2005) and high light intensity (Müller-Moulé et al. 2004; Talla et al. 2011). These studies on mutant and/or transgenic plants (summarized in the Table 1) with altered endogenous ASA levels proved that ascorbate plays a significant role in plant growth and development as well as abiotic stress tolerance. In this article, an attempt has been made to illustrate the role of ascorbate in various abiotic stresses in crop plants by exploring transgenic technology.

**Table 1:** **Role of ascorbate in plant growth and development and abiotic stress tolerance**

Enzyme/protein	Target plant	Gene	Gene source	Type of genetic manipulation	Ascorbate content	Phenotypic changes	Reference
GDP-mannose pyrophosphorylase	Tobacco	*GMPase*	Tomato	Overexpression	2.0–4.0-fold increase	Increased tolerance to temperature stress	Wang et al. 2011
Phosphomannose Isomerase	*Arabidopsis*	*PMI1*	*Arabidopsis*	RNAi	0.47–0.65-fold decrease	No phenotypic changes under normal growth conditions in both mutants	Maruta et al. 2008
		*PMI2*	–	T-DNA knockout	No change		
Phosphomannomutase	Tobacco	*NbPMM*	Tobacco	VIGS	Up to 3.0-fold decrease	–	Qian et al. 2007
	*Arabidopsis*	*NbPMM*	Tobacco	VVMEE	0.2–0.5-fold increase	–	
		*AtPMM*	*Arabidopsis*	Overexpression	0.25–0.33-fold increase	Increased tolerance to MV stress	
	Tobacco	*PMM*	Acerola	Overexpression	2.0-fold increase	–	Badejo et al. 2009
VTC4/Myoinositol monophosphatase (IMP)	*Arabidopsis*	*VTC4*	–	T-DNA knockout	0.61–0.75-fold decrease	22.4% –34% decreases in myoinositol content	Torabinejad et al. 2009
						Slow seed germination under control conditions	
						Slightly hypersensitive to ABA and NaCl during seed germination	
GDP-L-galactose phosphorylase	*Arabidopsis*	*vtc5-1* and *vtc5-2*	*Arabidopsis*	T-DNA knockout	0.2-fold decrease	Plant growth retardation and bleaching of the cotyledons	Dowdle et al. 2007
L-Galactose dehydrogenase	Tobacco (BY–2 cells)	*L–GalLDH*	Tobacco	Overexpression	1.5–2.0-fold increase	Higher mitotic index in cells	Tokunaga et al. 2005
						Reduced browning and cells death of cultures	
						Increased tolerance to MV	
L-galactono-1,4-lactone dehydrogenase	Tobacco (BY–2 cells)	*GLDH*	Tobacco	Antisense downregulation	0.30-fold decrease	Adversely effected plant cell division, growth and structure of plant cell	Tabata et al. 2001
	Tobacco	*RrGalLDH*	*Rosa roxburghii*	Overexpression	2.1-fold increase	Enhanced tolerance to salt stress	Liu et al. 2013a
Monodehydroascorbate reductase	Tobacco	*AtMDAR1*	*Arabidopsis*	Overexpression	Up to 2.2-fold increase	Enhanced tolerance to ozone, salt and PEG stresses	Eltayeb et al. 2007
	Tobacco	*Am-MDAR*	*Avicennia marina*	Overexpression	Up to 2.0-fold increase	Increased tolerance to salt stress	Kavitha et al. 2010
	Tobacco	*MDAR-OX*	*Arabidopsis*	Overexpression	Up to 1.1-fold increase	No change in Aluminium tolerance	Yin et al. 2010
Dehydroascorbate reductase	Tobacco	*DHAR-OX*	*Arabidopsis*	Overexpression	Up to 1.3-fold increase	Increased tolerance to Al stress	Yin et al. 2010
	Tobacco	*DHAR*	*Arabidopsis*	Overexpression	1.9–2.1-fold increase	Enhanced tolerance to ozone, drought and salinity	Eltayeb et al. 2006
	Tobacco	*DHAR*	Wheat	Overexpression	2.1-fold increase	Increased ozone tolerance and NPR	Chen and Gallie 2005
			Tobacco	Antisense downregulation	0. 29-fold decrease	Substantially reduced stomatal area and low NPR	
	Tobacco	*DHAR*	Human	Overexpression	No significant change	Enhanced tolerance to low temperature and NaCl	Kwon et al. 2003
Ascorbate peroxidase	Tobacco	*tAPx*	Tobacco	Overexpression	No change	Increased tolerance to MV and chilling stresses under light conditions	Yabuta et al. 2002
			Tobacco/ Spinach	Antisense downregulation	–	Plants failed to grow	
	*Arabidopsis*	*HvAPX1*	Barley	Overexpression	–	Increased tolerance to salt stress	Xu et al. 2008
	*Arabidopsis*	*OsAPXa and OsAPXb*	Rice	Overexpression	–	Increased tolerance to salt stress	Lu et al. 2007
	Tobacco	*CAPOA1*	Pepper	Overexpression	–	Increased plant growth	Sarowar et al. 2005
						Increased tolerance to MV stress	
	Tobacco BY-2 cells	*cAPX*	*Arabidopsis*	Antisense downregulation	No change	Increased tolerance against heat and salt stresses	Ishikawa et al. 2005
	Tobacco	*StAPX*	Tomato	Overexpression	–	Improved seed germination	Sun et al. 2010a
						Increased tolerance to salt and osmotic stresses	
	Rice	*Apx1/ Apx2*	Rice	RNAi (Apx1+ Apx2)	Up to 1.5-fold decrease	No change in plant growth and development	Rosa et al. 2010
						Increased tolerance to aluminium	
				RNAi (Apx1 or Apx2)	–	Produced semi-dwarf phenotype	
	Rice	*OsAPx-R*	Rice	RNAi	–	Delayed plant development	Lazzarotto et al. 2011
	Rice	*OsAPXa*	Rice	Overexpression	–	Increased spikelet fertility under cold stress	Sato et al. 2011
	Rice	*Osapx2*	Rice	Overexpression	–	Enhanced stress tolerance	Zhang et al. 2013
						Sensitive to abiotic stresses	
			–	T-DNA knockout	–	Semi-dwarf seedlings, yellow-green leaves, leaf lesion-mimic and seed sterility	
	Alfalfa	*Osapx2*	Rice	Overexpression	–	Increased salt resistance	Guan et al. 2012
	Tomato	*cAPX*	Pea	Overexpression	–	Enhanced tolerance to UV-B and heat stresses	Wang et al. 2006
	Tomato	*cAPX*	Pea	Overexpression	–	Enhanced tolerance to chilling and salt stresses	Wang et al. 2005
	Tomato	*LetAPX*	Tomato	Antisense downregulation	No significant change	Transgenic plants photosynthetically less efficient and sensitive to chilling stress	Duan et al. 2012b
Ascorbate oxidase	Tobacco	*AAO*	Cucumber	Overexpression	No change	Plants become susceptible to ozone	Sanmartin et al. 2003
	Tobacco	*AAO*	Cucumber	Overexpression	No change	Increased drought tolerance due to reduced stomatal conductance	Fotopoulos et al. 2008
	Tobacco	*AAO*	Pumpkin	Overexpression	2.0-fold increase in apoplastic ASA	Number of smaller flowers significantly increased 6% to 14% reduction of in seed weight	Pignocchi et al. 2003
			Tobacco	Antisense downregulation	2.0-fold increase in apoplastic ASA	No significant changes	
	Tobacco	*AAO*	Tobacco	Overexpression	–	Severe inhibition of germination and seed yield under high salinity	Yamamoto et al. 2005
	Tobacco	*AAO*	Tobacco	Antisense downregulation	–	Increased tolerance to salt stress	Yamamoto et al. 2005
					–	Increased seed yield under salt stress	
	*Arabidopsis*	*AAO*	–	T-DNA knockout		Increased tolerance to salt stress	
						Increased seed yield under salt stress	
Myoinositol oxygenase	Rice	*OsMIOX*	Rice	Overexpression	No change	Increased drought tolerance	Duan et al. 2012a
ASA mannose pathway regulator 1	*Arabidopsis*	*AMR1*	–	T-DNA knockout	2.0–3.0-fold increase	Increased ozone tolerance	Zhang et al. 2009

APx-R, APX-related; *CAPOA1*, *Capsicum annuum* ascorbate peroxidase-like 1 gene; MV, methyl viologen; NPR, net photosynthetic rate; PEG, polyethylene glycol; RNAi, RNA interference; VIGS, Virus-induced gene silencing; VVMEE, Viral-vector-mediated ectopic- expression.

## Overview: ascorbic acid biosynthesis, transportation, recycling and degradation processes in plants

In plants, the accumulation or steady level of ascorbate is maintained in homeostasis through the rate of synthesis, recycling and degradation, as well as intra- and inter-cellular transport (Horemans et al. 2000a; Pallanca and Smirnoff 2000; Green and Fry 2005).

### Biosynthesis

**Figure 1:**
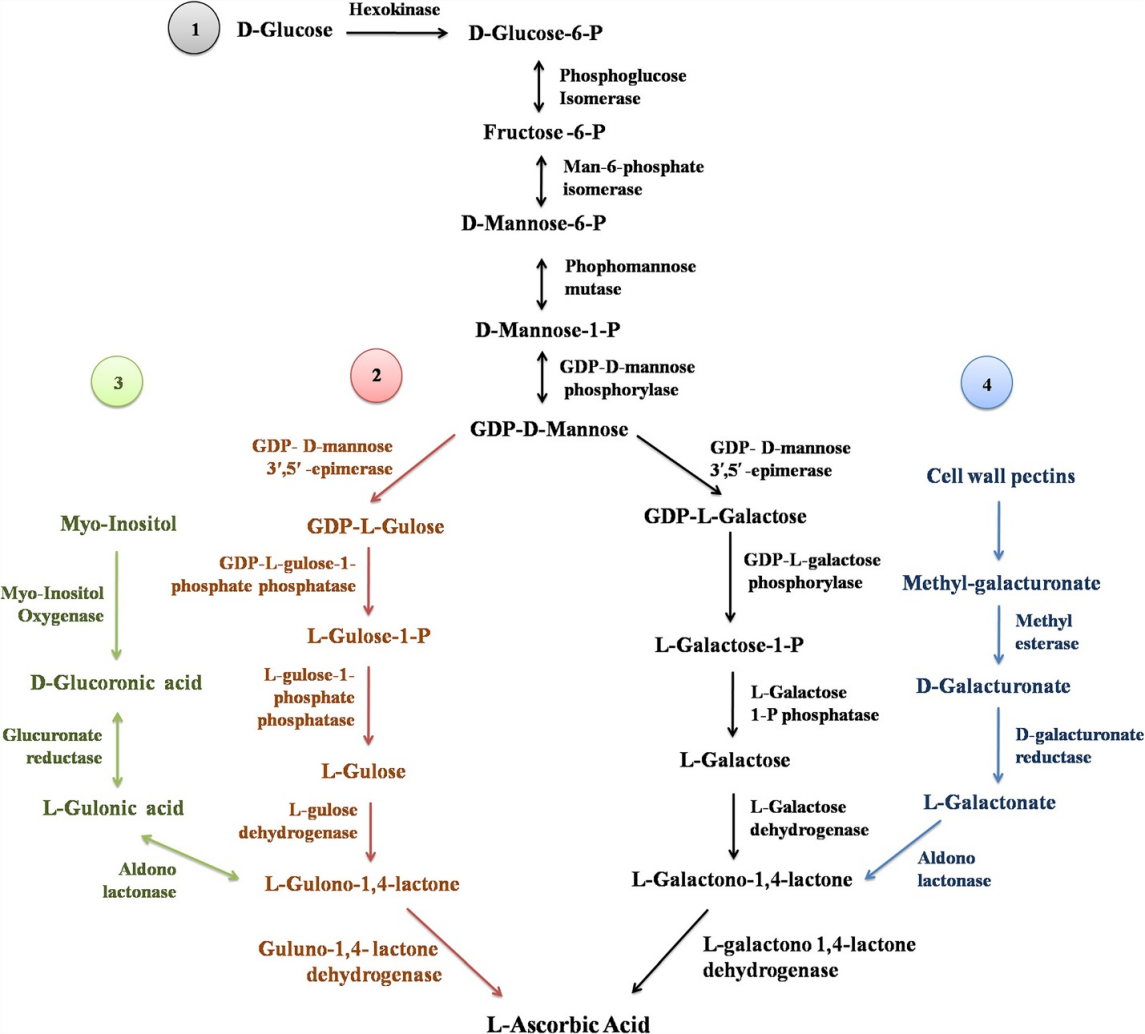
**L-ascorbic acid biosynthesis pathways in plants (modified after Hemavathi et al.** 2010 **): (1) Smirnoff-Wheeler pathway, (2) L-gulose pathway, (3) Myoinositol-based pathway, (4) D-galacturonic acid pathway.**

Characterization of low ascorbate producing mutants (*vtc*) of *Arabidopsis* has helped us to better understand the essential role of enzymes involved in the biosynthesis of L-ascorbate (Conklin et al. 1996; Conklin et al. 2000; Huang et al. 2005; Conklin et al. 2006; Müller-Moulé 2008). Now it is well known that in higher plants, ascorbate biosynthesis occurs through well-characterized D-mannose/L-galactose pathway (Smirnoff-Wheeler pathway), where D-mannose is converted to L-galactose via GDP-sugar intermediates (Wheeler et al. 1998) (Figure 1). L-galactose is further oxidized to L-galactono-1,4-lactone, which is converted into ascorbate, by L-galactono-1,4-lactone dehydrogenase (L-GalLDH), located on the inner mitochondrial membrane (Siendones et al. 1999; Smirnoff 2001). All of the genes that are involved in this pathway have been well-characterized; these include genes encoding GDP-D-mannose pyrophosphorylase (Conklin et al. 1999), GDP-D-mannose-3’,5’-epimerase (Wolucka and Van Montagu 2003; Watanabe et al. 2006), GDP-L-galactose phosphorylase (L-galactose guanylyltransferase) (Dowdle et al. 2007; Linster and Clarke 2008), L-galactose-1-phosphate phosphatase (Laing et al. 2004a), L-galactose dehydrogenase (Gatzek et al. 2002; Laing et al. 2004b) and L-GalLDH (Imai et al. 1998; Siendones et al. 1999; do Nascimento et al. 2005; Tokunaga et al. 2005; Alhagdow et al. 2007).

In addition to the Smirnoff-Wheeler pathway, three other potential pathways of ascorbate biosynthesis have been identified in plants. It was demonstrated that in addition to production of GDP-L-galactose, GDP-D-mannose-3’,5’-epimerase can also produce GDP-L-gulose (Davey et al. 1999; Wolucka and Van Montagu 2003). Moreover, exogenous L-gulose and L-gulono-1,4-lactone were shown to serve as direct precursors of ascorbate in *Arabidopsis* cell cultures (Davey et al. 1999). These observations led to a proposal for an alternative L-gulose pathway in which L-gulose and L-gulono-1,4-lactone are important intermediates (Wolucka and Van Montagu 2003). However, the intermediate steps in this pathway have not yet been characterized in plants. D-galacturonic acid pathway involves the conversion of D-galacturonic acid, a product of the degradation of cell wall pectins to L-ascorbate via L-galactono-1,4-lactone (Agius et al. 2003; Cruz-Rus et al. 2011; Badejo et al. 2012) (Figure 1). Following the cloning of *Arabidopsis* myoinositol oxygenase (MIOX) gene by Lorence et al. (2004), a myoinositol-based pathway (animal-like pathway) was proposed (Figure 1). MIOX converts myoinositol to D-glucuronate and plants can catalyze the conversion of D-glucuronate into L-gulonic acid. However, recently, Endres and Tenhaken (2009), proved that the MIOX is involved mainly in the modulation of the metabolite level of myoinositol and plays a negligible role in the plant ascorbate biosynthesis.

### Ascorbate transport

Once the ascorbate is synthesized on the inner mitochondrial membrane, it is transported to different cellular compartments including the apoplast. Both the ascorbate and DHA transport is mainly mediated by facilitated diffusion or active transport systems (Ishikawa et al. 2006). In contrast to ascorbate, DHA tends to be more efficiently transported across plant membranes with a higher affinity and capacity (Horemans et al. 1998; Szarka et al. 2004). It was proposed that specific plasma membrane transporters transport ASA or DHA in plants (Horemans et al. 2000b). However, either the protein or the gene associated with this transport and the nature of the mechanisms driving these carrier proteins are still inconclusive. Several other putative ascorbate transporters are associated with the plant plasma membrane (reviewed in Horemans et al. 2000a); however, the specific mechanisms by which they transport ASA or DHA have not been well elucidated.

Ascorbate biosynthesis occurs in almost all plant cells and tissues. However, its level is generally high in photosynthetic tissues, meristematic tissues, flowers, young fruits, root tips, and apices of stolons or tubers (Gest et al. 2013). In certain fruits, such as *Ribes nigrum* (by galactose pathway, Hancock et al. 2007) and strawberry (by D-galacturonic acid pathway, Agius et al. 2003), increased accumulation of ascorbate occurs by a combination of long-distance transport and in situ biosynthesis. High ascorbate demand in developing sink tissues is probably because it is critical for cell cycle and cell division/growth, which cannot be met entirely by sink tissue alone (Smirnoff 2000; Franceschi and Tarlyn 2002). Ascorbate accumulation in sink tissue is controlled to some extent by ascorbate biosynthesis in source tissues (Franceschi and Tarlyn 2002; Tedone et al. 2004). Franceschi and Tarlyn (2002), demonstrated that the long-distance transport of ASA in plants occurs via phloem, where L-ascorbate was found to be loaded into the phloem of source leaves and transported to sink tissues. In addition, ascorbate biosynthesis, which occurs in phloem tissue via the D-Man/L-Gal pathway could also contribute to ASA accumulation in plant storage organs (Hancock et al. 2003).

In mammals, sodium-dependent ascorbate transporters (SVCT1 and SVCT2), which belong to the nucleobase-ascorbate transporter (NAT) family, have been identified and well characterized as an active ascorbate transport system (Daruwala et al. 1999; Tsukaguchi et al. 1999; Ishikawa et al. 2006). Although numerous NATs have been identified in plants (Li and Schultes 2002; Maurino et al. 2006), their role in ASA transportation has not been established. Further studies are required to determine the definitive role in plant ascorbate transportation.

### Ascorbate recycling

ASA pool in cells is maintained through synthesis, recycling and transportation, and plays an important role in adaptation of plant to various stresses (Stevens et al. 2008). Ascorbate takes part in several enzymatic and non-enzymatic mechanisms for elimination of deleterious ROS (Asada and Takahashi 1987), and as a result, MDHA and DHA accumulates in the cells. The two enzymes involved in the oxidation of ascorbate are ascorbate oxidase (AAO) and ascorbate peroxidase (APX). AAO is an apoplastic enzyme that catalyzes the oxidation of ASA to MDHA using oxygen and is associated with cell wall metabolism and cell expansion (Smirnoff 1996). Ascorbate peroxidase (APX) is a class I peroxidase catalyzes the conversion of H_2_O_2_ into H_2_O, using ascorbate as a specific electron donor, thus resulting in the accumulation of MDHA as a by-product (Teixeira et al. 2004).

The ASA pool size is dependent, on both the rate of synthesis and the rate of reduction of MDHA and DHA back to ascorbate. MDHA and DHA produced as a result of activities of APX and AAO, respectively, should be efficiently recycled to maintain the reduced pool of ASA. MDHA is reduced back to ASA by MDAR using NADH/NADPH as electron donors. In addition, plant PM cyt b 561 (plasma membrane b-type cytochrome *c*) is also associated with the recycling of ASA from MDHA on the cytoplasmic side of the plasma membrane (Trost et al. 2000; Asard et al. 2001; Pignocchi and Foyer 2003). DHA is reduced to ASA by dehydroascorbate reductase (DHAR) using reduced glutathione (GSH) as an electron donor or by the electron-transport chain (ETC.) electron carriers (Szarka et al. 2007). Thus, DHAR and MDAR are crucial components in the maintenance of the reduced pool of ASA and are of prime importance in oxidative stress tolerance (Eltayeb et al. 2006).

### Ascorbate degradation

Although the pathway of ascorbate synthesis is distributed between the cytosol and the mitochondrion (Foyer 2004; Smirnoff et al. 2004), the ascorbate degradation pathway appears to reside in the apoplast (Green and Fry 2005). In most plants, ascorbate degradation can occur via dehydroascorbate, yielding oxalate (OxA) and L-threonate (ThrO). However, in some plants (Vitaceae eg. grape), ascorbate can also be degraded via L-idonate to L-threarate (L-tartrate) (Green and Fry 2005). A degradation pathway for ASA/DHA catabolism in plants has been reported recently (Simpson and Ortwerth 2000; Parsons and Fry 2012). Ascorbate degradation pathway involves enzymic and/or non-enzymic oxidation to dehydroascorbic acid (DHA), which may irreversibly hydrolyze to 2,3-diketogulonate (DKG). However, many of the enzymes involved in the degradation pathway of ASA are not well characterized in plants. Both DHA and DKG prone to further oxidation under the same physiological conditions as that of apoplast (Parsons and Fry 2012). DHA can be oxidized by H_2_O_2_ non-enzymatically to a monoanion (cyclic-oxalyl-threonate; cOxT) and a dianion (oxalyl-threonate [OxT] isomers, 3-OxT and 4-OxT) independently through formation of a reactive intermediate cyclic-2,3-O-oxalyl-L-threonolactone (Parsons et al. 2011). In the absence of H_2_O_2_, DKG is relatively stable, however slowly generates a range of products, such as 2-carboxy-l-xylonolactone, 2-carboxy-l-lyxonolactone and 2-carboxy-l-threo-pentonate (Parsons et al. 2011). In the presence of apoplastic plant esterases or prolonged non-enzymatic incubations, substantial hydrolysis of cOxT to OxT and then OxT to OxA and ThrO would take place (Parsons et al. 2011).

Genetic modulation of plant ascorbate pathway has become feasible with advancements made in plant genomics and genetic engineering. Several possible strategies have been followed to increase ascorbate production in plants via genetic engineering of enzymes involved in the biosynthesis and recycling of ascorbate. Several transgenes, which are of plant and animal origins, have been successfully used for increasing biosynthesis of ascorbic acid. Mouse L-gulono-c-lactone oxidase (*GLOase*) gene in tobacco, lettuce and potato (Jain and Nessler 2000; Hemavathi et al. 2010), human dehydroascorbate (*DHAR*) gene in tobacco (Kwon et al. 2003), wheat *DHAR* gene in tobacco and maize (Chen et al. 2003; Naqvi et al. 2009), *Arabidopsis* MDAR gene (*AtMDAR1*) in tobacco, strawberry D-galacturonic acid reductase (*GalUR*) gene in *Arabidopsis* and potato (Agius et al. 2003; Hemavathi et al. 2009) and rice L-GalLDH gene in rice (Liu et al. 2011) have been successfully cloned and expressed (summarized in the Table 2).

**Table 2:** **Transgenic approaches for overproduction of L-ascorbate in plants**

Enzyme	Target plant	Gene	Gene source	Type of genetic manipulation	Ascorbate content	Phenotypic change	Reference
GDP-l-galactose phosphorylases	Tomato	*GGP/ VTC2*	*Actinidia chinensis*	Overexpression	3.0–6.0-fold increase in fruits	–	Bulley et al. 2012
	Strawberry			Overexpression	2.0-fold increase in fruits	–	
	Potato		Potato/*Arabidopsis*	Overexpression	Up to 3.0-fold increase in tuber	–	
GDP-mannose pyrophosphorylase	Potato	*GMPase*	Potato	Antisense downregulation	0.88–1.44-fold reduction in leaves	Dark spots on leaf veins and stems	Keller et al. 1999
					0.56-fold reduction in tubers	Early senesce	
GDP-Mannose 3’,5’-epimerase	Tomato	*SlGME1*	Tomato	Overexpression	Up to 1.42-fold increase in leaves	Improved tolerance to various abiotic stresses such as cold, salt and MV	Zhang et al. 2011
					Up to 1.60-fold increase in fruits		
		*SlGME2*		Overexpression	Up to 1.37-fold increase in leaves		
					Up to 1.24-fold increase in fruits		
L-galactose guanyltransferase	Tobacco	*GalT*	Kiwifruit	Transient expression (leaves)	Up to 3.0-fold increase	–	Laing et al. 2007
L-Galactose dehydrogenase	Tobacco	*L-GalDH*	*Arabidopsis*	Overexpression	No change	–	Gatzek et al. 2002
	*Arabidopsis*		*Arabidopsis*	Antisense downregulation	0.7-fold decrease	–	
L-galactono-1,4-lactone dehydrogenase	Rice	*L-GalLDH*	Rice	RNAi	0.6–0.87-fold decrease	Slow plant growth rate and poor seed set	Liu et al. 2011
			Rice	Overexpression	Up to 1.48-fold increase	Increased NPR and higher seed set	
	Tomato	*SlGalLDH*	Tomato	RNAi	No change	Slow plant growth rate	Alhagdow et al. 2007
						Strong reduction in leaf and fruit size	
	Rice	*L-GalLDH*	Rice	RNAi	0.3– 0.5-fold decrease	Slow growth rate, reduced tiller number, decreased NPR and premature senescence	Liu et al. 2013b
L-gulono-c-lactone oxidase	*Arabidopsis*	*GLOase*	Rat	Overexpression	Up to 2.0–3.0-fold increase	–	Radzio et al. 2003
	Lettuce	*GLOase*	Rat	Overexpression	4.0–7.0-fold increase	–	Jain and Nessler 2000
	Tobacco			Overexpression	Up to 7.0-fold increase	–	
	Tomato	*GLOase*	Rat	Overexpression	1.5-fold increase in fruits	Enhanced tolerance to MV, NaCl, and mannitol	Lim et al. 2012
	Potato	*GLOase*	Rat	Overexpression	Up to 1.41-fold increase	Enhanced tolerance to MV, NaCl, and mannitol	Hemavathi et al. 2010
D-galacturonic acid reductase	*Arabidopsis*	*GalUR*	Strawberry	Overexpression	2.0–3.0-fold increase	–	Agius et al. 2003
	Potato	*GalUR*	Strawberry	Overexpression	1.6–2.0-fold increase	Enhanced tolerance to MV, NaCl, and mannitol	Hemavathi et al. 2009
	Tomato (Hairy Roots)	*GalUR*	Strawberry	Overexpression	2.5-fold increase	High growth rate	Wevar Oller et al. 2009
Monodehydroascorbate reductase	Tomato	*LeMDAR*	Tomato	Overexpression	Up to 1.18-fold increase	Enhanced tolerance to temperature (low/high) and MV stresses	Li et al. 2010
						High NPR	
				Antisense downregulation	Up to 1.3-fold decrease	Susceptible to various abiotic stresses	
	Tomato	*MDAR*	Tomato	Overexpression	0.7-fold reduced in fruits	–	Haroldsen et al. 2011
					No change in leaves		
Dehydroascorbate reductase	Tomato	*DHAR*	Tomato	Overexpression	1.6-fold increase in fruits	–	Haroldsen et al. 2011
					No change in leaves		
	Maize (Kernels)	*DHAR*	Wheat	Overexpression	6.0-fold increase	–	Naqvi et al. 2009
	Maize	*DHAR*	Wheat	Overexpression	Up to 1.8-fold (leaves) and 1.9–fold (kernels) increase	–	Chen et al. 2003
	Tobacco	*DHAR*	Wheat	Overexpression	2.2–3.9-fold increase	–	Chen et al. 2003
	Tobacco	*DHAR*	Rice	Overexpression	Up to 1.6-fold increase	Enhanced tolerance to salt and cold stresses	Le Martret et al. 2011
	Tobacco	*DHAR*	Human	Overexpression (chloroplasts)	1.1-fold increase	Increased SOD and APX activities in conjunction via triple gene construct	Lee et al. 2007
						Increased tolerance to MV and NaCl induced stress	
	Potato	*DHAR*	Sesame	Overexpression	1.1–1.3-fold increase in tuber with *patatin* promoter	–	Goo et al. 2008
				Overexpression	1.5- and 1.6-fold increase in leaves and tuber respectively, with *CaMV35S* promoter	1.5- and 1.6-fold increase in leaves and tuber respectively, with *CaMV35S* promoter	
	Potato	*StDHAR1*	Potato	Overexpression (Cytosol)	Up to 0.69-fold increase in leaves	–	Qin et al. 2011
					Up to 0.29-fold increase in tubers		
					Up to 0.50-fold increase in leaves	–	
		*StDHAR2*		Overexpression (Chloroplast)	No significant change in tubers		
	*Arabidopsis*	*DHAR1*	Rice	Overexpression	> 1.4-fold increase	Enhanced tolerance to salt stress	Ushimaru et al. 2006
	*Arabidopsis*	*DHAR*	*Arabidopsis*	Overexpression	2.0–4.25-fold increase	Enhanced tolerance to high–light and high–temperature stress	Wang et al. 2010
Myoinositol oxygenase	*Arabidopsis*	*miox4*	*Arabidopsis*	Overexpression	2.0–3.0-fold increase	–	Lorence et al. 2004

MV, methyl viologen; NPR, net photosynthetic rate; RNAi, RNA interference.

## Role of ascorbate in photosynthesis as a photoprotectant

**Figure 2:**
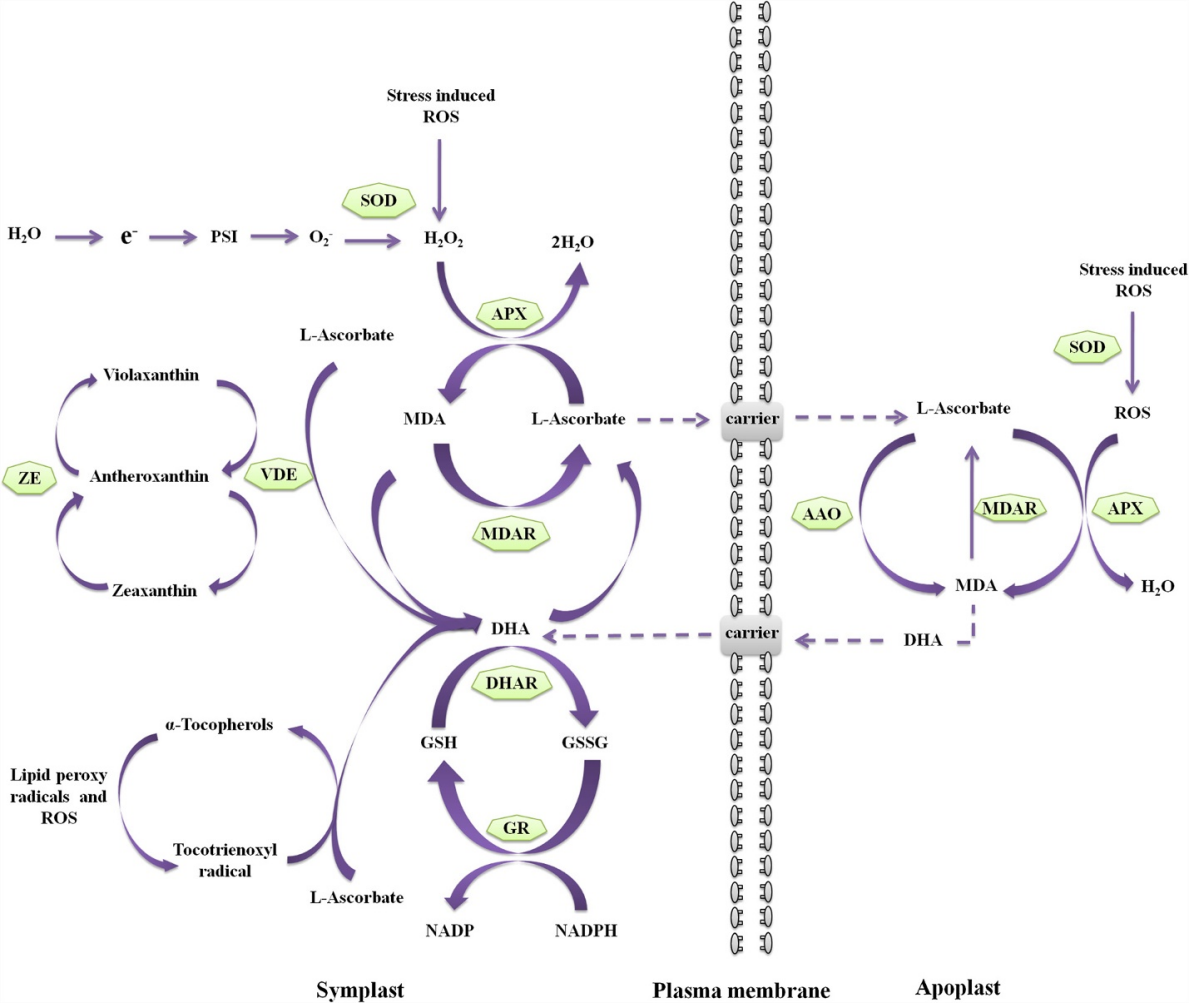
**Multiple functions of L-ascorbate in plants.** During abiotic stress conditions, scavenging of ROS by APX increases MDA content in both apoplast and symplast. If the MDA is not rapidly reduced back to ascorbate by MDAR, spontaneously disproportionate into ascorbate and DHA. Cytoplasmic DHAR can reduce DHA back to ascorbate using GSH, and the resulting GSSG is regenerated back to GSH through the action of GR in a NADPH dependent reaction. Furthermore, during oxidative stress conditions, L-ascorbate acts as a cofactor for violaxanthin de-epoxidase for the formation of zeaxanthin and also involves in the regeneration of α-tocopherol from tocotrienoxyl radicals.

A high concentration of ascorbate in chloroplasts would imply its central role in photosynthesis (Smirnoff 1996). Ascorbate plays a crucial roles in scavenging the deleterious ROS that are generated as by-products of photosynthesis and as a key component in excess photonic energy dissipation mechanisms, such as the water-water cycle (WWC) (Neubauer and Yamamoto 1992; Asada 1999) and the xanthophyll cycle (Müller-Moulé et al. 2002; Yabuta et al. 2007). WWC, which is also known as Mehler peroxidase reaction, is one of the most important detoxification systems functioning in intact chloroplasts (Asada 1994,1999,2006). It involves the photoreduction of O_2_ by PSI to a superoxide radical, followed by the dismutation of superoxide radical by superoxide dismutase (SOD) to hydrogen peroxide and oxygen (Müller-Moulé et al. 2002). The hydrogen peroxide is reduced to water by ascorbate, catalyzed by ascorbate peroxidase (APX), and the resulting by-product monodehydroascorbate (MDA) is directly reduced to ascorbate either by reduced ferredoxin of PSI (Miyake and Asada 1992; Miyake and Asada 1994; Asada 1999) or by NAD(P)H-dependent chloroplastic MDHA reductase using NADH or NADPH as electron donor (Sano et al. 2005). MDHA can spontaneously disproportionate to ascorbate and dehydroascorbate (DHA) (Asada 1999). DHA is unstable at the physiological pH and irreversibly degrade to 2,3 diketo-1-gulonic acid if not recycled back to ascorbate. To preserve the ascorbate pool, DHA should be rapidly reduced back to ascorbate. DHA is recycled back to ascorbate via the ascorbate-glutathione cycle by reduced glutathione (GSH), catalyzed by DHAR (Shimaoka et al. 2003). Finally, glutathione reductase (GR) converts glutathione disulfide (GSSG) back into GSH using NAD(P)H as a reducing agent (Figure 2). Recently, Huang et al. (2008), reported that thioredoxin *h2* (Trx *h* 2) having both DHA reductase (in the presence of glutathione) and MDA reductase (in the presence of NADH) activity may also involve in the regeneration of ascorbate from DHA and MDHA, respectively.

Increased biosynthesis of ascorbate in high light exposed plants and enhanced photoinhibition and oxidative damage in ascorbate-deficient plants would imply its role in excess light energy dissipation (Smirnoff 2000; Müller-Moulé et al. 2004; Yabuta et al. 2007). It was previously reported that high light stress results in the induction of the cytosolic APX and protects the cytosol and other cellular compartments from high light induced oxidative stress (Mittler 2002; Mullineaux and Karpinski 2002). Several isoforms of APX have been found in many plant species including both monocots and dicots, and are localized to various subcellular compartments. In *Arabidopsis*, nine *APX* genes were described (Panchuk et al. 2002; Mittler et al. 2004; Narendra et al. 2006; Koussevitzky et al. 2008): two cytosolic, two microsomal, three chloroplastic, one mitochondrial, and one dual-targeted to mitochondria and chloroplasts (Chew et al. 2003). In tomato, *APX* gene family comprises of seven genes encoding three cytosolic, two peroxisomal, and two chloroplastic APXs (Najami et al. 2008). Whereas, in rice, the *APX* gene family consists of eight genes encoding two cytosolic, two peroxisomal, and three chloroplastic isoforms and one is targeted to the mitochondria (Teixeira et al. 2004,2006; Hong et al. 2007). Recently, Lazzarotto et al. (2011), characterized a new class of rice putative heme peroxidases, APX-R (APX-related), a dually localized protein, targeted to both chloroplasts and mitochondria, which is functionally associated with APX. *APX* genes have been partially characterized in some plant species such as spinach (Ishikawa et al. 1995,1996,1998), cowpea (D’Arcy-Lameta et al. 2006) and eggplant (Lin et al. 2007). The large functional diversity and subcellular localization of the APX genes suggest the degree of complementation and coordination of the antioxidant defences in different cellular compartments during development and abiotic stress (Teixeira et al. 2004,2006).

APX is highly responsive to various abiotic stresses and plays an important role in the scavenging of ROS in plants. Mutant studies in *Arabidopsis* revealed that cytosolic APXs (APX1 and APX2) are critical for cellular H_2_O_2_ homeostasis and play an important role in growth, development and oxidative protection of chloroplasts under various abiotic stresses (Pnueli et al. 2003; Davletova et al. 2005; Koussevitzky et al. 2008; Zhang et al. 2013). In particular, *Arabidopsis* APX1 is important for plant growth and development (Pnueli et al. 2003), whereas APX2 is critical for drought tolerance (Rossel et al. 2006). Thylakoid-bound APXs (tAPXs) are crucial for photosynthesis and photoprotection under photo-oxidative stress in *Arabidopsis* (Kangasjarvi et al. 2008). In rice, expressions of *OsAPX* genes are modulated by various abiotic stresses and exogenous ABA as well as by biotic stresses (Agrawal et al. 2003; Teixeira et al. 2006; Hong et al. 2007; Rosa et al. 2010). The expressions of two cytosolic APX genes, *OsAPX1* and *OsAPX2*, are developmentally regulated (Agrawal et al. 2003) and the suppression of either of these genes resulted in strong effects on plant growth and development and produced semi-dwarf rice phenotypes (Rosa et al. 2010). Zhang et al. (2013), reported similar results wherein, downregulation of *OsAPX2* gene affected the growth and development of rice seedlings, resulting in semi-dwarf and lesion-mimic seedlings, yellow-green leaves, and seed sterility. In contrast, the overexpression of *OsAPX2* gene increased APX enzyme activity and thus resulted in enhanced stress tolerance.

Davletova et al. (2005), demonstrated the role of cytosolic APX1 in cross-compartment protection of thylakoid/stromal and mitochondrial APXs during light stress. Despite the protection of each individual cellular compartment by its own set of ROS-scavenging enzymes, APX1-deficient *Arabidopsis* plants exhibited the oxidation of chloroplastic, mitochondrial and membrane-bound proteins, suggesting the key role of cytosolic APX1 enzyme in the cross-compartment protection of adjacent compartments (Davletova et al. 2005). However, some early studies certainly suggest that thylakoid membrane-bound APX (tAPX) is a limiting factor of antioxidative systems under photo-oxidative stress in chloroplasts and that the enhanced activity of tAPX under stress is to maintain the redox status of ascorbate (Yabuta et al. 2002). Moreover, transgenic *Arabidopsis* plants overexpressing *Suaeda salsa* chloroplastic stromal APX (sAPX) and thylakoid-bound APX (tAPX) also showed an increased tolerance to high light oxidative stress by efficient detoxification of ROS (Pang et al. 2011).

Ascorbate also plays a significant role in formation of zeaxanthin during photo-oxidative stress (Figure 2). The excess excitation energy from the incidence of high light is invariably dissipated as heat by zeaxanthin in the light harvesting complex of the photosynthetic apparatus (Demmig-Adams and Adams 1996). Zeaxanthin is regenerated (via Xanthophyll cycle) in the successive de-epoxidation of violaxanthin and antheroxanthin by the enzyme VDE, which is located in the thylakoid lumen, and requires ascorbate as a cofactor (Neubauer and Yamamoto 1993; Müller-Moulé et al. 2002). Müller-Moulé et al. (2003), demonstrated the role of ascorbate in regeneration of zeaxanthin in ascorbate-deficient mutant of *Arabidopsis*, *vtc2*. These plants are characterized with an increased degree of lipid peroxidation and photoinhibition, and the regeneration of zeaxanthin from violaxanthin was slower due to insufficient ascorbate content.

## Role of l-ascorbate in salinity and drought tolerance

In the cell, ROS is continuously produced during normal functioning of the photosynthesis, respiration and photorespiration as well as in various enzyme-catalyzed redox reactions (Dat et al. 2000; Moller 2001). However, ROS activity increases several folds under stress conditions and can serve as a signal that activates defense responses by specific signal transduction pathway in which hydrogen peroxide acts as secondary messenger (Helena and de Carvalho 2008). However, an increased ROS activity for the prolonged period can cause oxidative stress in plants. If ROS is not efficiently scavenged and quenched, it can cause membrane lipid peroxidation, inactivation of cellular enzymes and degradation of nucleic acids, which may eventually lead to the death of plant cells.

Plants with higher ascorbate content can effectively scavenge the excessive ROS generated during stress conditions, and confer increased tolerance to abiotic stresses. Increased salt stress sensitivity of the *Arabidopsis vtc* mutant is attributed to the low intrinsic ascorbate levels and impaired ascorbate-glutathione cycle, which resulted in an enhanced ROS activity and a significant decrease in the CO_2_ assimilatory capacity (Huang et al. 2005). Moreover, deficiency of ascorbate may limit the recycling of α-tocopheroxyl radicals to α-tocopherol, which may, in turn, increase the oxidation of thylakoid membrane lipids under drought conditions (Munné-Bosch and Alegre 2002). Several transgenic plants overproducing ascorbate showed an enhanced salt and drought tolerance with reduced membrane lipid peroxidation and chlorophyll content loss. These plants also exhibited higher survival rate and a significantly higher seed germination rate, fresh weight and root length (Wang et al. 2005; Sun et al. 2010a; Zhang et al. 2011). Transgenic potato plants expressing strawberry *GalUR* gene and rat *GLOase* gene with several-fold increased biosynthesis of ascorbate also exhibited a better survival under salinity and drought stresses conditions including a reduction in the level of lipid peroxidation (Hemavathi et al. 2009; Hemavathi et al. 2011; Upadhyaya et al. 2011).

Regulation of plant ascorbate redox state by means of synthesis, degradation and transport plays an essential role in plant adaptation to the stress (Stevens et al. 2008; Yin et al. 2010). MDAR and DHAR are key enzymes involved in the regulation of the ascorbate redox state and are of vital importance in the oxidative stress tolerance. MDAR maintains higher redox state of ascorbate by recycling the oxidized MDHA. Several isoforms of MDAR have been found in different cellular compartments, such as chloroplasts (Miyake and Asada 1994; Sano et al. 2005), cytosol and mitochondria (De Leonardis et al. 1995; Jiménez et al. 1997; Mittova et al. 2003), peroxisomes (Mittova et al. 2003; Leterrier et al. 2005) and glyoxysomes (Bowditch and Donaldson 1990), to serve the specific physiological role in each cellular compartment. The level of MDAR expression increases in response to oxidative stress triggered by several stress conditions (Yoon et al. 2004; Leterrier et al. 2005; Kavitha et al. 2010). Transgenic tobacco plants overexpressing a salt-inducible chloroplastic MDAR from halophyte *Avicennia marina* survived better under conditions of salt stress compared with wild-type plants (Kavitha et al. 2010). Similarly, transgenic potato plants overexpressing the *Arabidopsis DHAR* gene in the cytosol exhibited enhanced DHAR activity with faster growth, even under drought and salt stress conditions (Eltayeb et al. 2011).

High salt and drought tolerances were also observed in transgenic plants overexpressing *APX* gene. Heterologous expression of *OsAPX2* gene improved salt tolerance in transgenic *Arabidopsis* and alfalfa (Lu et al. 2007; Guan et al. 2012). Increased APX activity was observed in roots of etiolated rice seedlings in response to NaCl stress and was correlated with upregulation of chloroplastic OsAPX8 expression; however, no effect on the expression of the rest of the rice APX isoforms was observed (Hong et al. 2007). In contrast, Teixeira et al. (2006), reported the enhanced expression of OsAPX2 and OsAPX7, and severe downregulation of OsAPX8 in rice seedlings under NaCl stress. This observed discrepancy in the above results seemed to be differ with cultivars, plant age, tissues, and growing conditions (Hong et al. 2007).

It has been demonstrated that *OsAPX* gene expression and H_2_O_2_ production were increased in response to NaCl in roots of etiolated rice seedlings (Tsai et al. 2004,2005). However, OsAPX8 expression and APX activity induced by NaCl are not mediated through H_2_O_2_ in rice roots (Tsai et al. 2005; Hong et al. 2007). In rice roots, accumulation of ABA in response to NaCl was correlated with upregulation of OsAPX8 expression (Hong et al. 2007). Moreover, exogenous application of ABA also specifically enhanced the expression of OsAPX8. Similarly, application of ABA increased the expression of APX genes in pea, rice, and sweet potato (Mittler and Zilinskas 1992; Agrawal et al. 2003; Park et al. 2004). These findings indicate that NaCl induced expression of APX is mediated through an accumulation of the ABA.

Transgenic plants overexpressing a heterologous cytosolic *APX* gene showed an enhanced tolerance to salt stress with lower ROS activity (Badawi et al. 2004; Wang et al. 2005; Lu et al. 2007; Faize et al. 2011). These transgenic plants exhibited lower electrolyte leakage and lipid peroxidation, higher water use efficiency, minimal leaf damage and better photosynthetic performance. Similar results were obtained in the transgenic tobacco overexpressing *Solanum lycopersicum* thylakoid-bound APX (tAPX) and showed a better performance in terms of photosynthetic efficiency, root lengths and fresh and dry weights of the plants with enhanced tolerance to salt and osmotic stresses (Sun et al. 2010a).

Yamamoto et al. (2005), demonstrated that downregulation of apoplastic AAO (ascorbate oxidase) confers higher salt tolerance in tobacco and *Arabidopsis* plants. It was suggested that under salt stress conditions, suppressed expression of apoplastic AAO led to a relatively low level of hydrogen peroxide accumulation and a high redox state of symplastic and apoplastic ascorbate, which, in turn, increased the salt tolerance. Interestingly, transgenic tobacco plants with elevated levels of hydrogen peroxide by overexpression of a cell wall-localized cucumber AAO conferred increased drought tolerance due to reduced stomatal conductance (Fotopoulos et al. 2008).

Control of the stomatal aperture is essential for the plant adaptation to changes in its ambient environment. Several mechanisms for the regulation of stomatal aperture have been proposed (Kim and Lee 2007; Araújo et al. 2011). It has been found that O_2_^-^ and other activated oxygen species are involved in the regulation of stomatal movement (Purohit et al. 1994). Zhang et al. (2001), demonstrated that hydrogen peroxide may function as an intermediate in ABA signalling in guard cells. During stress conditions ABA causes an increase in hydrogen peroxide production and induces stomatal closure. Stomatal closure induced by hydrogen peroxide was reversed by exogenous application of ascorbate because of hydrogen peroxide detoxification activity of ascorbate (Zhang et al. 2001). Earlier, Chen and Gallie (2004), demonstrated that transgenic plants with DHAR overexpression exhibited an increase in the ascorbate redox state and reduced levels of hydrogen peroxide in guard cells and leaves showed greater stomatal opening, increased transpiration rate and stomatal conductance even under normal growth conditions. Whereas, plants with suppression of DHAR activity showed an elevated level of hydrogen peroxide and conferred increased drought tolerance with a decreased ascorbate redox state.

It has been known that the enzyme AAO, which catalyzes the oxidation of ASA to DHA exclusively located in the apoplast, plays an important role in the maintenance of the redox state of the apoplastic ascorbate levels (Pignocchi and Foyer 2003; Sanmartin et al. 2003; Pignocchi et al. 2006). However, the mechanism of regulation of *AAO* gene expression and stomatal moments is not clearly understood. It has been suggested that the signal perception of stomatal closure is altered by AAO overexpression (Pignocchi and Foyer 2003; Fotopoulos et al. 2008). Transgenic tobacco leaves overexpressing a cell wall-localized cucumber AAO contained elevated levels of hydrogen peroxide and ABA content, thereby resulting in reduced stomatal conductance and reduced rates of water loss (Fotopoulos et al. 2008). Based on these results, it is predictable that either the suppression of DHAR expression or the overexpression of AAO would result in the decrease in the ascorbate redox state and causes increased accumulation of hydrogen peroxide levels resulting in stomatal closure, lower transpiration thus providing drought tolerance. However, in both, suppression of DHAR expression or overexpression of AAO would result in greater accumulation of apolastic DHA levels which may play a key role in the regulation of stomatal aperture.

## Ascorbate as an ozone protectant

An increasing concentration of ambient ozone was observed during recent decades in many industrial and rural regions of the world and poses a hazard for vegetation. The ozone exposure of plants causes extensive visible leaf damage and decreased rates of stomatal conductance and photosynthesis (Pell et al. 1997; Torsethaugen et al. 1997; Zheng et al. 2000; Sanmartin et al. 2003). Ozone entered through stomata reacts with apoplastic and symplastic components of the cell (Long and Naidu 2002; Castagna and Ranieri 2009; Cho et al. 2011) resulting in a greater accumulation of ROS, which causes an oxidative damage to the photosynthetic membranes and finally leads to the death of photosynthetic mesophyll cells (Godde and Buchhold 1992; Ciompi et al. 1997; Chen et al. 2005). It was suggested that ozone exposure directly affects guard cells by inhibiting the ion channels (K^+1^ channel) activity in the guard cell plasma membrane (Torsethaugen et al. 1999). Protection of crop plants from ozone damage could be accomplished by replacement of sensitive biotypes with more tolerant ones as well as by application of synthetic ozone protectants such as ethylene diurea, azoxystrobin, epoxiconazole and penconazole (Blum et al. 2011; Didyk and Blum 2011). However, application of synthetic ozone protectants will pollute the environment and may affect the crop production. Therefore, it is necessary to develop alternative ecofriendly strategies to minimize the ozone damage in plants by using plant-based natural antioxidants such as ascorbic acid.

Apoplastic ascorbate is assumed to represent the first line of defence against potentially damaging pollutants (Plöchl et al. 2000; Barnes et al. 2002). Apoplastic ascorbate can protect plants from ozone-induced damage by directly reacting with ozone (Chameides 1989; Plöchl et al. 2000) and ROS (D’Haese et al. 2005) or by serving as a substrate in ROS-scavenging enzymatic reactions (Chen and Gallie 2005). Plant species that are resistant to ozone showed an increased apoplastic ascorbate levels (Lee 1991; Turcsányi et al. 2000; Zheng et al. 2000; Burkey et al. 2006; Feng et al. 2010). Moreover, exogenous application of plants with ascorbate prevented the foliar injury and alleviated the decline in photosynthesis rate caused by ozone stress (Maddison et al. 2002; Zheng et al. 2000). The lower levels of apoplastic ascorbate content greatly enhanced foliage injury upon chronic ozone exposure in tobacco (Sanmartin et al. 2003). Furthermore, *Arabidopsis* mutants (*vtc1*) with low foliar content of ascorbate exhibit hypersensitivity to ozone (Conklin and Barth 2004). Similarly, rice TOS17 insertional mutant (ND6172) for a GDP-D-mannose-3’,5’-epimerase gene, which is characterized with 20–30% lower ascorbate level than the wild type, showed a higher level of visible leaf damage upon ozone exposure (Frei et al. 2012).

Maintenance of the apoplastic ascorbate redox state is crucial for ozone-induced oxidative stress tolerance of plants and is influenced by activities of enzymes such as AAO and APX. Altered expression of these enzymes was normally observed in plants exposed to ozone (Kubo et al. 1995; Sanmartin et al. 2003; Pignocchi et al. 2006). Transgenic tobacco plants with overexpressing AAO (Sanmartin et al. 2003) or downregulation of cytosolic APX (Orvar and Ellis 1997) resulted in the increased susceptibility of tobacco plants to ozone-induced damage. However, transgenic tobacco plants overproducing chloroplastic APX could not protect from ozone injury (Torsethaugen et al. 1997). The apoplastic ascorbate redox state also depends on the balance between oxidation of ascorbate to DHA in apoplast and reduction of MDA and DHA to ascorbate in cytoplasm. During the detoxification process, DHA produced in the apoplast diffuses into the cytoplasm and recycled back to ascorbate by cytDHAR (via ascorbate-glutathione cycle, Figure 2) on the plasma membrane. The regenerated ascorbate can be transported back into the apoplast for the detoxification of ozone (Luwe et al. 1993; Horemans et al. 2000a; Yoshida et al. 2006). Transgenic tobacco plants overexpressing *MDAR* gene conferred enhanced tolerance to ozone due to increased recycling of ascorbate from MDA (Eltayeb et al. 2007). Similarly, DHAR-overexpressing plants also showed an increased ozone tolerance with a higher level of photosynthetic activity despite exhibiting a larger stomatal area (Chen and Gallie 2005). In converse, plants with suppressed DHAR activity showed a substantially reduced stomatal area and lower level of photosynthetic activity. Yoshida et al. (2006), demonstrated that *Arabidopsis* mutant with completely lacking cytDHAR activity showed a significantly lower level of apoplastic ascorbate and was highly sensitive to ozone (Yoshida et al. 2006). Increased level of ascorbate through enhanced ascorbate recycling by DHAR overexpression offered greater protection against oxidative stress despite poor ability to respond to ozone through stomatal closure (Chen and Gallie 2005; Eltayeb et al. 2007).

## Role of ascorbate in temperature stress tolerance

Temperature stress is one of the most important environmental factors affecting the crop yields and geographic distribution of plants. Temperature stresses such as heat, cold or freezing result in excessive ROS production and cause severe damage to cell membranes and proteins (O’Kane et al. 1996; Larkindale and Knight 2002; Suzuki and Mittler 2006; Hu et al. 2008; Yamashita et al. 2008) and also cause impairments in the chloroplast and mitochondrial metabolism (Salvucci and Crafts-Brandner 2004; Vacca et al. 2004; Barra et al. 2005; Nguyen et al. 2009; Barta et al. 2010; Tóth et al. 2011).

Several studies demonstrated that ROS-mitigating mechanisms play an important role in protecting crops against extreme temperature stresses (Iba 2002; Yoshimura et al. 2004; Hu et al. 2008). For instance, overexpression of cytosolic APX in transgenic tomato enhances heat and chilling stress tolerance (Wang et al. 2005,2006). Similarly, transgenic potato plants overexpressing APX under the control of an oxidative stress inducible *SWPA2* promoter showed increased tolerance to high temperature stress (Tang et al. 2006). In rice, overexpression of OsAPX1 enhanced tolerance to chilling stress at the booting stage (Sato et al. 2011). Increased temperature stress tolerance was also observed in transgenic tobacco plants overexpressing the thylakoid-bound *APX* gene from tomato. These transgenic tobacco lines, under stress condition, showed a higher APX activity and contained less hydrogen peroxide and malondialdehyde than wild-type plants (Sun et al. 2010b). Moreover, under chilling and heat stresses, the photochemical efficiency of PSII in the transgenic lines was distinctly higher than that of wild-type plants. Wang et al. (2011), reported the similar results in transgenic tobacco plants overproducing ascorbate through the expression of tomato GMPase and observed the reduced ROS activity in the transgenic plants under high or low temperature stress conditions.

L-ascorbate may also act as an alternative electron donor of PSII; in those cases electron transfer is inhibited due to inactivation of oxygen evolving complex (OEC) (Mano et al. 2004; Guiss´e et al. 1995; Strasser 1997; Tóth et al. 2009; Gururani et al. 2012). Heat-induced inactivation of PSII was strongly influenced by the ascorbate content of leaves (Tóth et al. 2011). Tóth et al. (2011), experimentally proved the physiological role of ascorbate as alternative PSII electron donor in heat-stressed leaves with inactive OEC. This result suggests that the role of ascorbate as an alternative PSII electron donor is to decelerate the processes of photoinactivation and minimize the ROS activity in the photosynthetic thylakoid membranes, and thus minimize the damage to the entire photosynthetic apparatus.

## Conclusion

In higher plants, ascorbate biosynthesis occurs through D-mannose/L-galactose pathway, which is a most important source of ascorbate. Ascorbate plays a major role in cellular ROS-scavenging activity. It also influences many stress responsive enzyme activities through synergic action with the other antioxidants such as glutathione and α-tocopherol and reduces the oxidative damage to cells. Recent studies suggest its role in photosynthesis as an alternative electron donor to PSII under abiotic stress conditions and play a major role in protection of photosynthetic apparatus in chloroplast by keeping the ROS activity under check.

Several ascorbate biosynthetic pathway transgenes have been introduced into plants through genetic engineering to elevate the ascorbate level. These transgenic plants also provided better stress tolerance to various abiotic stresses such as high light, low/high temperature, ozone, salinity and drought. The role of ascorbate goes beyond that of simply an antioxidant given its apparent involvement in a complex signalling pathway that mediates responses to biotic and abiotic stresses as it is a cofactor for plant hormones such as ABA, GA and ethylene (Conklin and Barth 2004). However, role of ascorbate in signal transduction needs to be clarified further, particularly with respect to drought tolerance provided by altered stomatal movements.

## Authors’ original submitted files for images

Below are the links to the authors’ original submitted files for images. Authors’ original file for figure 1 Authors’ original file for figure 2

## Abbreviations

ABA: Abscisic acid
AGC: The ascorbate-glutathione cycle
AAO: Ascorbate oxidase
APX: Ascorbate peroxidase
DHA: Dehydroascorbate
DHAR: Dehydroascorbate reductase
GA: Gibberellic acid
GalUR: D-galacturonic acid reductase
GLOase: L-gulono-c-lactone oxidase
GR: Glutathione reductase
GSH: Glutathione
GSSG: Glutathione disulfide
MDAR: Monodehydroascorbate reductase
MDHA: Monodehydroascorbate
MIOX: Myoinositol oxygenase
L-GalLDH: L-galactono-1,4-lactone dehydrogenase
OEC: Oxygen evolving complex
PSI: Photosystem I
PSII: Photosystem II
SOD: Superoxide dismutase
ROS: Reactive oxygen species
VDE: Violaxanthin de-epoxidase
WWC: The water-water cycle.
