# Supplementary figures and images for: Role of L-ascorbate in alleviating abiotic stresses in crop plants
Source: Bot Stud. 2014 Apr 9;55:38. doi: 10.1186/1999-3110-55-38 (PMC5432849; doi:10.1186/1999-3110-55-38)

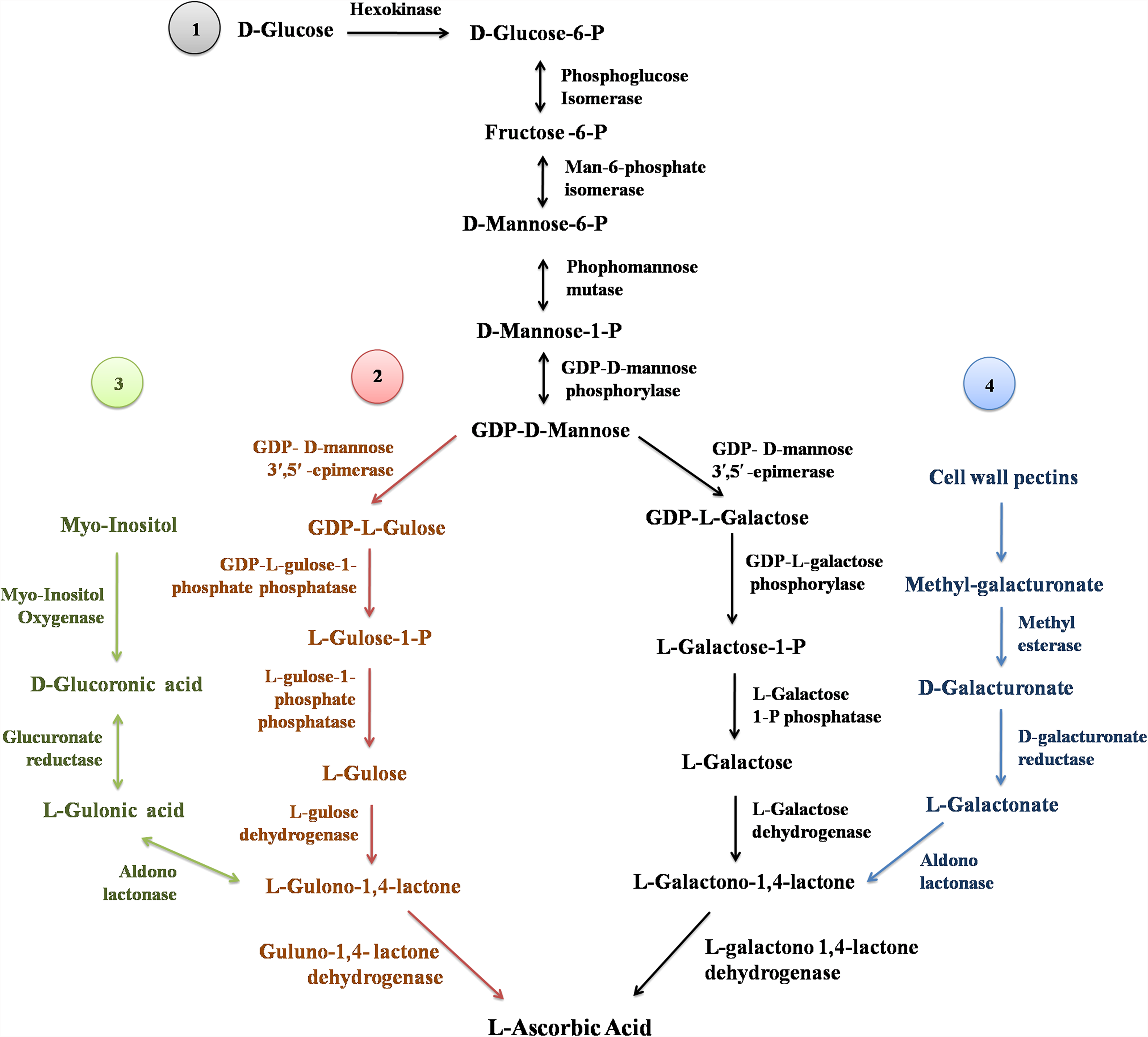

Supplement: Supplementary file 1 — Authors’ original file for figure 1 [file 40529_2012_90_MOESM1_ESM.tif]

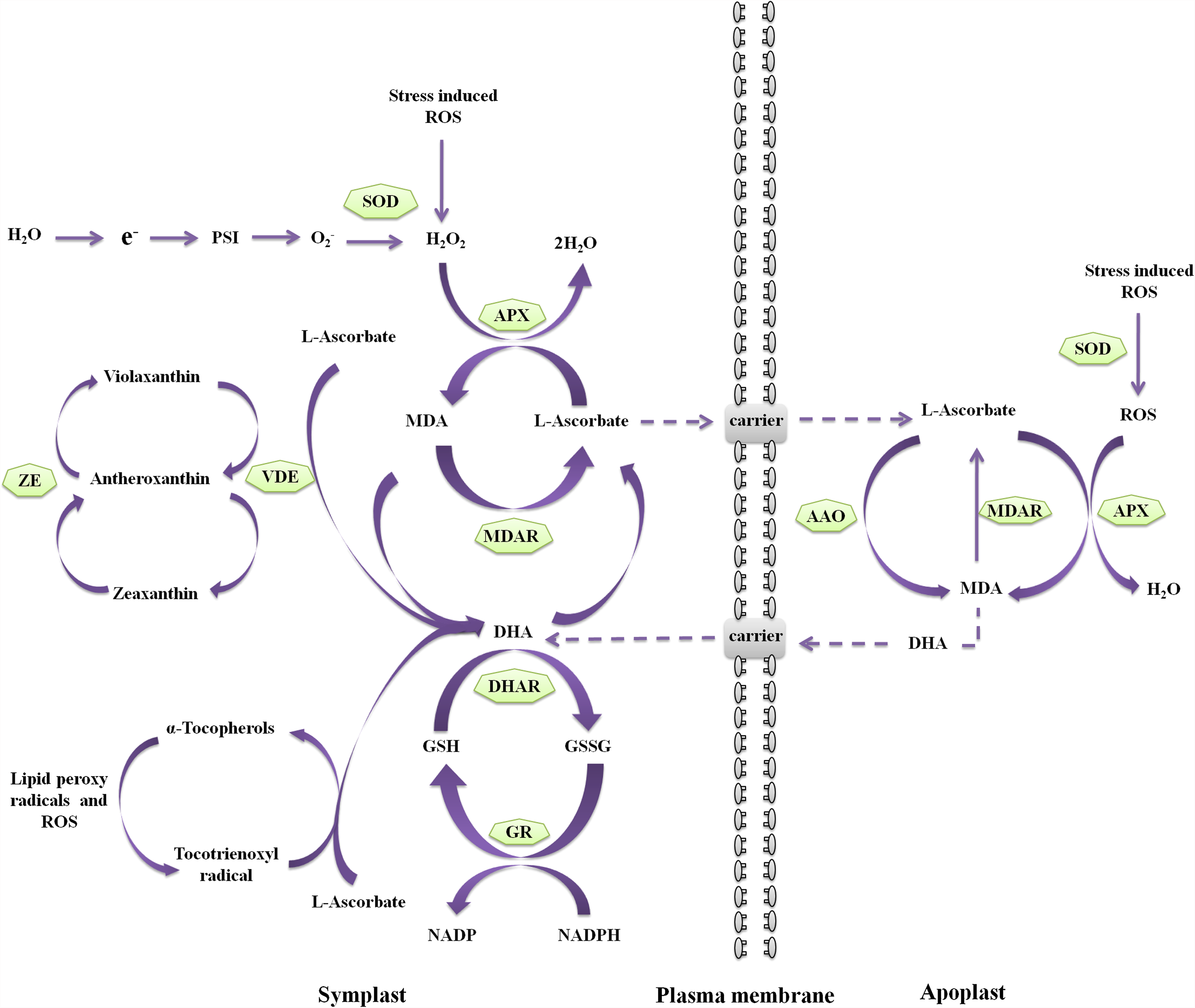

Supplement: Supplementary file 2 — Authors’ original file for figure 2 [file 40529_2012_90_MOESM2_ESM.tif]
